# Partial Portal Vein Arterialization Attenuates Acute Bile Duct Injury Induced by Hepatic Dearterialization in a Rat Model

**DOI:** 10.1155/2016/7427246

**Published:** 2016-10-30

**Authors:** Jun Jiang, Jishu Wei, Junli Wu, Wentao Gao, Qiang Li, Kuirong Jiang, Yi Miao

**Affiliations:** Department of General Surgery, The First Affiliated Hospital of Nanjing Medical University, 300 Guangzhou Road, Nanjing, Jiangsu Province 210029, China

## Abstract

Hepatic infarcts or abscesses occur after hepatic artery interruption. We explored the mechanisms of hepatic deprivation-induced acute liver injury and determine whether partial portal vein arterialization attenuated this injury in rats. Male Sprague-Dawley rats underwent either complete hepatic arterial deprivation or partial portal vein arterialization, or both. Hepatic ischemia was evaluated using biochemical analysis, light microscopy, and transmission electron microscopy. Hepatic ATP levels, the expression of hypoxia- and inflammation-associated genes and proteins, and the expression of bile transporter genes were assessed. Complete dearterialization of the liver induced acute liver injury, as evidenced by the histological changes, significantly increased serum biochemical markers, decreased ATP content, increased expression of hypoxia- and inflammation-associated genes and proteins, and decreased expression of bile transporter genes. These detrimental changes were extenuated but not fully reversed by partial portal vein arterialization, which also attenuated ductular reaction and fibrosis in completely dearterialized rat livers. Collectively, complete hepatic deprivation causes severe liver injury, including bile infarcts and biloma formation. Partial portal vein arterialization seems to protect against acute ischemia-hypoxia-induced liver injury.

## 1. Introduction

Hepatic artery (HA) thrombosis causes serious biliary complications such as nonanastomotic biliary strictures and bile leakage in liver transplant recipients [1, 2]. However, outside the liver transplantation setting, the HA is believed to be expendable since the liver receives an abundant blood supply from the proper HA, the portal vein (PV), and extensive collateral pathways in the native liver [3]. Previous animal experiments have demonstrated that HA ligation alone does not result in histological and biochemical abnormalities [4, 5]. HA occlusions such as HA ligation, transarterial embolization, and transcatheter arterial chemoembolization are widely applied clinically. Nevertheless, many cases of hepatic infarction and liver abscesses following hepatic arterial insufficiency have been reported. Thomas et al. reported the occurrence of hepatic necrosis after HA ligation during hilar cholangiocarcinoma resection [6]. Furthermore, right HA injury during laparoscopic cholecystectomy has caused liver infarcts or abscesses [7, 8]. The incidence of posttransarterial embolization and posttranscatheter arterial chemoembolization liver abscess is 0.26% [9] and 0.2%–4.5% [10], respectively. In liver transplant recipients, the incidence of liver abscess is 11.5% [11] and the underlying mechanisms are not clear.

Unlike hepatic parenchymal cells, the biliary tree is exclusively supplied by a network of arterioles and capillaries stemming from the hepatic arterial branches, namely, the peribiliary vascular plexus (PBP) [12]. In addition, cholangiocytes are more susceptible to arterial ischemia than hepatocytes [2, 13]. It is possible that ischemic bile injury plays a role in liver abscess but the mechanisms are as yet not elucidated. Hence, it was hypothesized that ischemic bile duct injury contributes to the development of liver abscess after HA deficiency.

Some researchers propose that PV arterialization should be applied with caution due to its negative effects [14, 15]; therefore it is now mainly used in acute hepatic failure, HA, or PV thrombosis after liver transplantation and extensive hepatobiliary surgery [14, 16]. Considering the salutary effect of partial portal vein arterialization (PPVA) on hepatocytes [17, 18], it was hypothesized that PPVA could also protect cholangiocytes from acute ischemic injury.

The effects of complete hepatic deprivation in a rat model of bile duct ischemia (BDI) were explored to determine whether PPVA had a protective effect on acute ischemic bile duct injury in this model.

## 2. Materials and Methods

### 2.1. Animals

Male Sprague Dawley rats (180–260 g) were purchased from Shanghai Laboratory Animal Center (Shanghai, China). The animals were kept under controlled environmental conditions with a 12 h light/dark cycle and fed commercial rat chow and tap water ad libitum. All experiments were conducted in compliance with the Guide for the Care and Use of Laboratory Animals and were approved by the Animal Care and Use Committee of Nanjing Medical University.

### 2.2. Surgical Procedures

**Figure 1:**
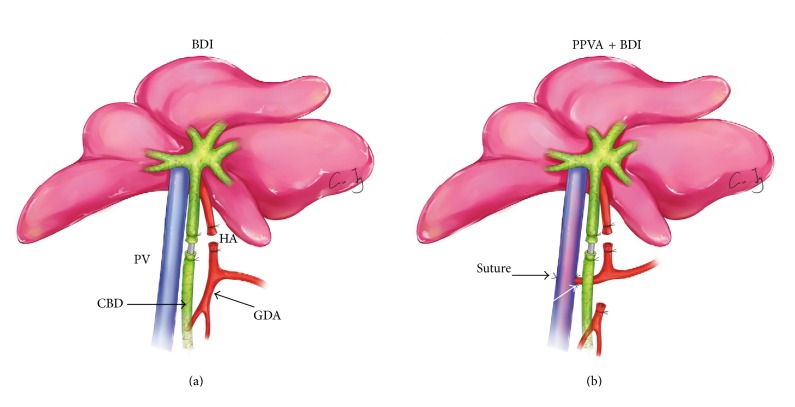
Surgical procedures. (a) Bile duct ischemia (BDI) was induced as follows: The hepatic artery (HA) was ligated and divided. The common bile duct (CBD) was cannulated with a polyethylene tube and doubly ligated to interrupt the peribiliary plexus. (b) BDI was followed by partial portal vein arterialization (PPVA), which was performed as follows: The gastroduodenal artery (GDA) was divided. A piece* *of* *silk* *suture with a small needle was reserved at the proximal end* *of* *the GDA. The* *dorsal common HA and the left edge of* *the portal vein (PV) wall were sutured together using two pieces of 8-0 prolene sutures (white arrow). The reserved needle along with the GDA was guided between the two sutures* *into* *the PV lumen* *and* *out from* *the* *opposite* *side. The GDA retracted into the PV after the surplus end of the GDA was excised. Hemostasis was achieved using a suture (black arrow). The PV was clamped for <5 min during the whole procedure.

All rats were anesthetized with sodium pentobarbital (50 mg/kg, intraperitoneally). After laparotomy, the common bile duct (CBD) and HA were mobilized, and the peripheral collateral vessels were dissected as previously described [19]. Rats were randomized (6–8 animals per group) to the following groups: (1) Sham control group: no further intervention was performed. (2) PPVA group: PPVA was performed as previously described, with some modifications [20]. Briefly, the HA and gastroduodenal artery (GDA) were mobilized, doubly ligated, and divided. A piece* *of* *silk* *suture with a small tapercut needle was reserved at the proximal end* *of* *the GDA. The* *dorsal common HA and the left edge of* *the PV wall were sutured together using two pieces of 8-0 prolene sutures. After the PV was clamped with a microvascular clamp, the tapercut needle with the GDA was guided between the two sutures* *into* *the PV lumen and* *out from* *the* *opposite* *side, where an 8-0 prolene suture was deployed for further hemostasis. After the surplus end of the GDA was excised, the GDA was retracted into the PV. Once fresh blood was observed to* *flow out of the PV wound, the prepositioned prolene suture was tightened. Tremor of the PV could* *be* *detected after the microvascular clamp was removed. The total occlusion time was <5 min. (3) BDI group: BDI was established as previously reported [4]. Briefly, the HA was doubly ligated and divided. The CBD was cannulated with a polyethylene tube (PE-10, 0.28 mm inner diameter, 0.64 mm outer diameter, American Health & Medical Supply International Corp. Co. Ltd., New York, NY, USA) and doubly ligated to interrupt the PBP (Figure 1(a)). (4) BDI + PPVA group: the BDI procedure was followed by PPVA (Figure 1(b)).

To determine the early and late effects of hepatic deprivation, animals were sacrificed on postoperative days 1 or 3 and 14. Some rats were randomly sampled for ultrasonographic examination. Blood samples collected from the PV for gas analysis were immediately analyzed using a portable blood gas analyzer (i-STAT, Abbott, Illinois, USA). Blood samples from the inferior vena cava were centrifugated at 4°C and separated plasma was stored at −80°C for further biochemical analysis. The liver was fixed in 4% neutral buffered formaldehyde or 2.5% glutaraldehyde for further analysis. Serum and liver tissue samples from six control rats served as the baseline samples.

### 2.3. Portal Vein Pressure and Biochemical Analysis

During relaparotomy, the patency of the GDA-PV anastomosis was checked using a high-frequency ultrasound apparatus (Vevo2100, VisualSonics, Canada) with a 16 MHz transducer. A 25-gauge needle attached to a transducer was introduced into the PV to measure the pressure. Serum aspartate aminotransferase (AST), alkaline phosphatase (AKP), bile acid, and total bilirubin levels were measured using standard analytical methods.

### 2.4. Histological Analysis

Liver tissues were fixed in 4% neutral buffered formaldehyde, paraffin-embedded, sectioned (4 *μ*m), and stained with hematoxylin and eosin or Sirius Red using standard histological techniques. To quantify the activity of bile duct proliferation, bile ducts in 10 portal tract-centered areas were counted in high magnification (200x). In some rats, India ink (Oddfoni Co. Ltd., Nanjing, China) was injected into the CBD at 8 mL/h, as previously reported [21] before histological examination. Collagen deposition was determined by measurement of liver hydroxyproline content as previously described [22]. Liver samples were measured with a spectrophotometer at 570 nm wavelength based on a standard curve.

### 2.5. Transmission Electron Microscopy

Transmission electron microscopy (TEM) was performed as previously described [21]. Briefly, liver tissue was fixed in 2.5% glutaraldehyde in cacodylate buffer and 1% OsO_4_, infiltrated with acetone-araldite, and embedded in araldite. Next, 80 nm sections were treated with uranyl acetate and lead nitrate before examination using a JEM-1010 electron microscope (JEOL Ltd., Japan).

### 2.6. Adenosine Triphosphate Measurements

The hepatic adenosine triphosphate (ATP) content was measured using a firefly luciferase-based ATP assay kit (Beyotime, Nantong, China) according to the manufacturer's instructions. Briefly, liver samples were homogenized and lysed with the lysis buffer in the kit and centrifuged at 12,000 ×g for 10 min at 4°C. Then, 100 *μ*L supernatant was mixed with 100 *μ*L monitoring reagent. Luminescence was measured using the Dual-Luciferase Reporter Assay System (Promega, E1910, WI, USA). Standard curves were generated. Protein concentration was determined using a bicinchoninic acid assay (BCA) kit (Keygen, Nanjing, China).

### 2.7. Immunohistochemistry

Immunohistochemical analysis was performed as previously described [23]. The primary antibody used was mouse monoclonal antihypoxia inducible factor-1*α* (anti-HIF-1*α*, Sigma, St. Louis, MO, USA) and antialpha smooth muscle actin (anti-*α*-SMA, Abcam, Cambridge, MA, USA). Histological images were captured using a Nikon Eclipse 80i microscope equipped with a Nikon Digital Sight DS-U3 microscope camera controller (Nikon, Japan). Quantification of *α*-SMA staining was assessed by the ratio of the area of yellow staining to the total area using Image-Pro Plus Version (6.0).

### 2.8. Quantitative Real-Time Polymerase Chain Reaction

Quantitative real-time polymerase chain reaction (qRT-PCR) was performed as previously described [23]. Briefly, total RNA was extracted from 50 mg liver tissue using the TRIzol reagent (Invitrogen, Shanghai, China), and cDNA was synthesized using the PrimeScript RT kit (Takara, Dalian, China). QRT-PCR was performed using FastStart Universal SYBR Green Master (Rox, Roche, USA) on an ABI PRISM 7500 Sequence Detection System (Applied Biosystems, Life Technologies Corp., USA). The relative mRNA expression of HIF-1*α*, vascular endothelial growth factor (VEGF), Na^+^ taurocholate cotransporting peptide (Ntcp,* SLC10A1*), bile salt excretory pump (Bsep,* ABCB11*), tumor necrosis factor- (TNF-) *α*, interleukin- (IL-) 1*β*, and procollagen I was calculated as the inverse log of ΔΔCt and normalized to the reference gene, *β*-actin. All experiments were performed in triplicate. The primers (see Table 1) were synthesized by Invitrogen.

### 2.9. Western Blotting

Total liver protein was prepared as previously reported [23]. The protein concentration was determined using a BCA kit. Proteins were separated using 10% sodium dodecyl sulfate polyacrylamide gel electrophoresis and transferred to polyvinylidene difluoride membranes (Millipore Corporation, Billerica, MA, USA). The membranes were blocked in 5% nonfat dried milk and incubated overnight at 4°C with the primary antibodies including mouse monoclonal anti-HIF-1*α* antibody (Sigma), rabbit polyclonal anti-Bsep and anti-Ntcp antibodies (Abcam), and mouse monoclonal TNF-*α* antibody (R&D Systems Inc., Minneapolis, MN, USA). GAPDH antibody (Beyotime) was used as an internal control. Electrochemiluminescence was performed with a ChemiImager 5500 imaging system (Alpha Innotech Co., San Leandro, CA, USA).

### 2.10. Myeloperoxidase Activity

Liver samples were homogenized in five volumes of normal saline and centrifuged at 3000 rpm at 4°C for 10 min. Myeloperoxidase (MPO) levels in the supernatant were measured using a commercial assay kit (Nanjing Jiancheng Bioengineering Institute, Nanjing, China) following the manufacturer's instructions.

### 2.11. Statistical Analysis

The results are expressed as mean ± SEM. All statistical analyses were conducted using SPSS 13.0 for Windows (SPSS, Chicago, IL, USA). Data were compared between multiple groups by two-way analysis of variance (ANOVA). Comparisons between pairs were performed by Bonferroni posttests. *P* < 0.05 was considered statistically significant.

## 3. Results

### 3.1. PPVA Extenuated BDI-Induced Biochemical Changes

**Figure 2:**
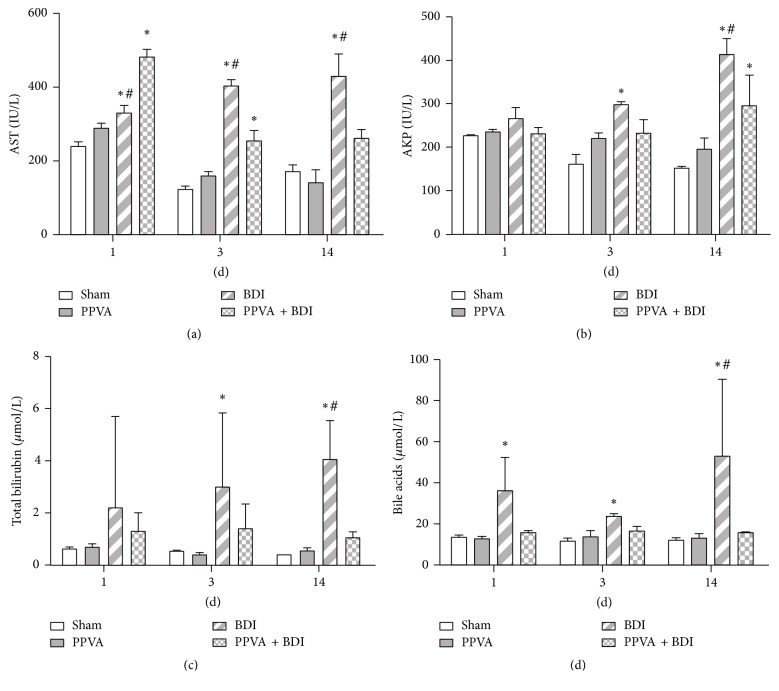
PPVA extenuated BDI-induced biochemical changes. Blood samples were subjected to biochemical analysis for (a) AST, (b) AKP, (c) total bilirubin, and (d) bile acids. Data are expressed as mean ± SEM (6–8 rats per group). ^*∗*^ *P* < 0.05 versus the Sham group; ^#^ *P* < 0.05 versus the PPVA + BDI group. Differences were assessed by two-way ANOVA and comparisons between groups and at each time point were performed by Bonferroni posttests.

Serum AST, AKP, total bilirubin, and bile acid levels were significantly increased in the BDI group. These changes were extenuated in the PPVA + BDI group (Figure 2). In this group, serum AST levels were significantly elevated on postoperative day 1 and then decreased (Figure 2(a)), indicating that PPVA might transiently aggravate hepatocyte damage. No significant difference in biochemical profiles was detected between the PPVA and Sham groups.

### 3.2. PPVA Improved BDI-Induced Bile Duct Injury

**Figure 3:**
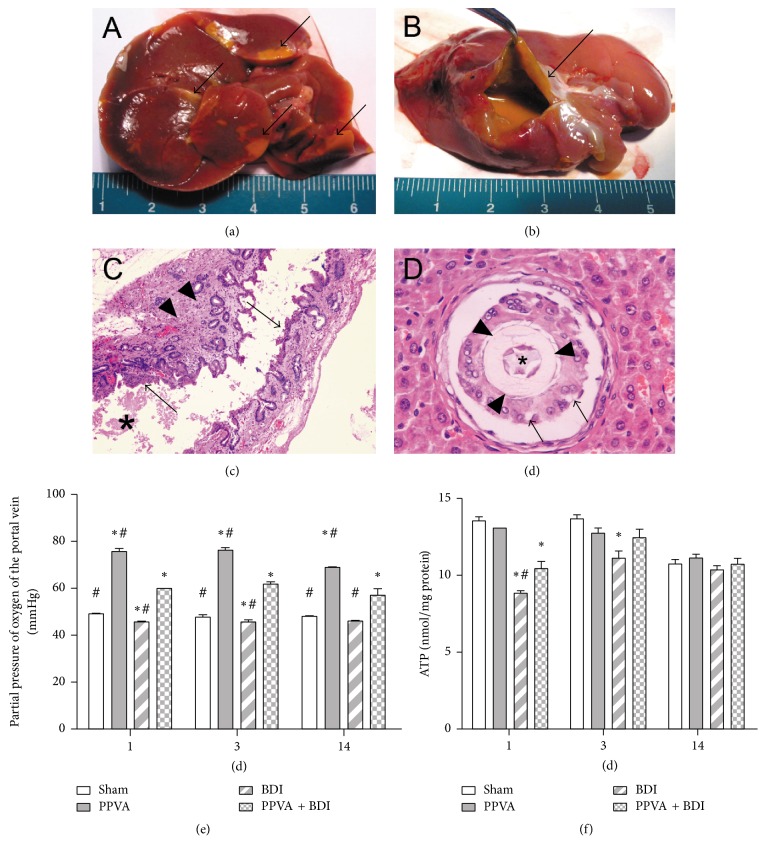
BDI-induced bile duct injury. Representative gross photographs of (a) bile leakage and (b) biloma formation after BDI. (c) Hematoxylin and eosin (H&E) staining shows desquamated biliary epithelium (arrows), biliary casts (asterisk), and fibrosis (arrowheads) 14 days after the surgery (magnification, 100x). (d) Biliary casts (asterisk) were also detected in the lumen of the cannulation tube (arrowheads). Cholangiocyte vacuolization was observed (arrows; H&E staining, magnification, 400x). (e) PPVA improved partial pressure of oxygen of the portal vein. (f) PPVA ameliorated BDI-induced ATP depletion. Data are expressed as mean ± SEM (6 rats per group). ^*∗*^ *P* < 0.05 versus the Sham group; ^#^ *P* < 0.05 versus the PPVA + BDI group. Differences were assessed by two-way ANOVA and comparisons between groups were performed by Bonferroni posttests at each time point.

In the BDI group, bile infarcts with mild inflammatory cell infiltration, bile leakage, and biloma formation were exclusively found in some completely dearterialized livers (Figures 3(a) and 3(b)). Epithelial desquamation and fibrosis in large bile ducts were detected in the BDI group on light microscopy (Figures 3(c) and 3(d)). The rats in the PPVA + BDI group did not show these changes (data not shown).

### 3.3. PPVA Improved BDI-Induced Dysfunction of Energy State

The oxygen tension in the PV was investigated. PPVA improved the hypoxia situation in the PPVA + BDI group compared with the BDI group, as evidenced by the elevated partial pressure of oxygen in the PV (Figure 3(e)). Consistently, ATP synthesis was significantly decreased on days 1 and 3 in the BDI group, compared with the Sham control group. PPVA improved the energy state, as shown by an elevation in ATP content (Figure 3(f)).

### 3.4. Mechanism of Bile Leakage

**Figure 4:**
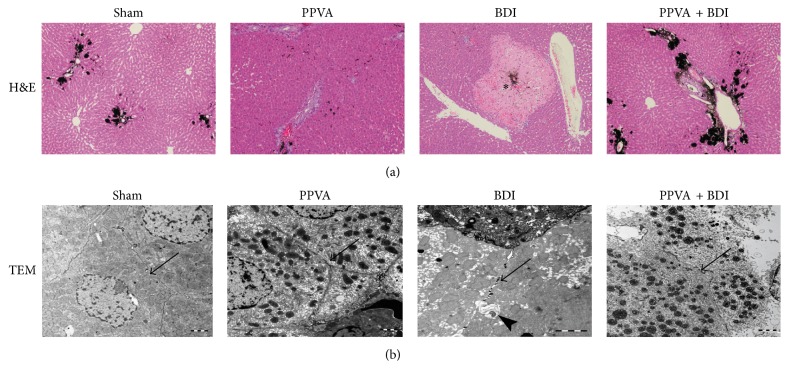
PPVA protected completely dearterialized rat livers against necrosis 3 days after surgery. (a) The relationship between the biliary system and bile infarcts was assessed by injecting India ink into the CBD at a low pressure. The ink was detected in the centers of bile infarcts (asterisk), indicating that the biliary system communicated with the infarcts (H&E staining; magnification, 100x). (b) Coagulative necrosis of hepatocytes was detected in the BDI group on transmission electron microscopy. The tight junctions (arrows) and bile canaliculi (arrowhead) between necrotic hepatocytes were intact and not dilated (scale bars, 2 *μ*m).

To investigate the relationship of the biliary system with the bile infarcts, India ink was injected into the CBD at a low pressure. Normally, India ink deposition in periportal areas and bile canaliculi is detected on histological analysis. In the BDI group, India ink deposits were surrounded by bile infarcts, indicating that the biliary system communicated with the infarcts (Figure 4(a)). TEM revealed coagulative necrosis of cholangiocytes and hepatocytes and intact tight junctions and no dilatation in necrotic hepatocytes (Figure 4(b)). This, at least partly, indicates that bile may leak from necrotic bile canaliculi or ductules.

### 3.5. PPVA Attenuated BDI-Induced Ductular Reaction and Fibrosis

**Figure 5:**
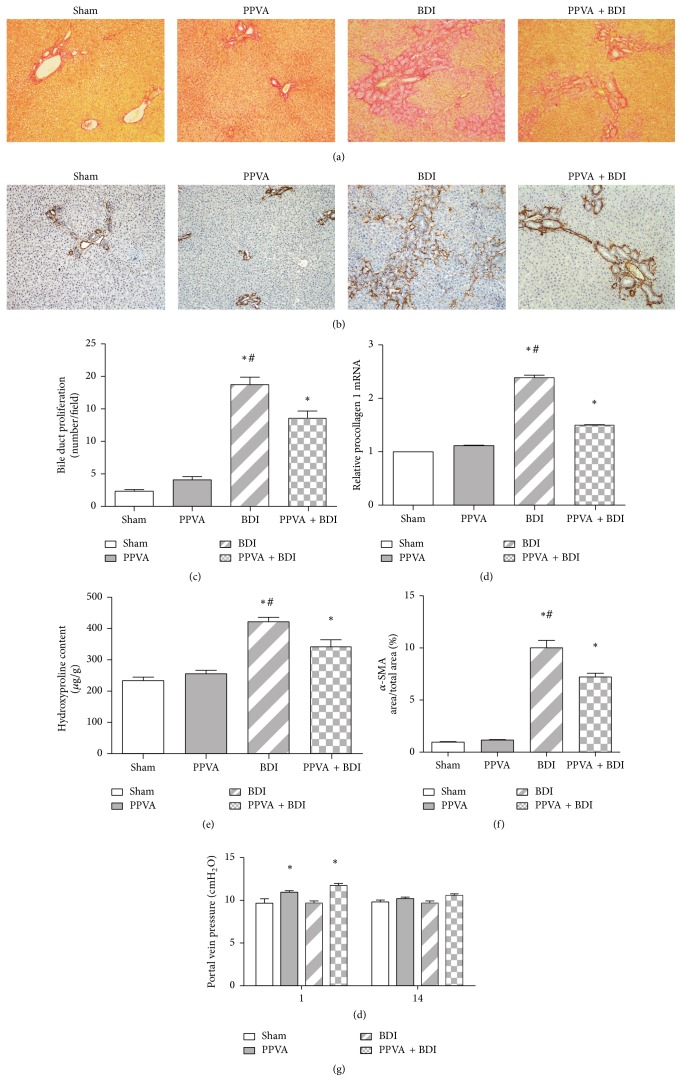
PPVA reduced BDI-induced ductular reaction and fibrosis. Representative microscopic images of Sirius Red staining (a) and alpha-smooth muscle actin (*α*-SMA) immunostaining (b) are shown 14 days after surgery (magnification, 100x). (c) The magnitude of the ductular reaction was reduced by PPVA (bile ducts in 10 portal tract-centered areas were counted in high magnification in each group). (d) Procollagen I mRNA expression was evaluated using qRT-PCR. (e) Collagen content was evaluated by measurement of liver hydroxyproline. (f) Quantification of *α*-SMA immunostaining area was accessed by Image-Pro Plus software. (g) PV pressure was measured in all groups to examine whether PPVA induced PV hypertension. PCR results are expressed as the mean ± SEM of at least triplicate measurements. ^*∗*^ *P* < 0.05 versus the Sham group; ^#^ *P* < 0.01 versus the PPVA + BDI group. Differences were assessed by two-way ANOVA and comparisons between groups were performed by Bonferroni posttests at each time point.

Increased bile duct proliferation and mild inflammatory cell infiltration in portal areas were detected in the BDI group 3 days after surgery (data not shown), but they were not significant. In parallel with the ductular reaction, fibrotic deposits were detected around the reactive ductules from day 3 and had progressed by day 14 in the BDI group (Figure 5(a)). Ductular reaction and fibrosis were significantly reduced by PPVA (Figures 5(a)–5(c)). In the Sham- and PPVA-operated rats, liver sections showed minimal physiologic changes with little fibrosis, which was mostly confined to the portal areas. There was a significant collagen deposition in both the BDI group and the PPVA + BDI group compared with the Sham group (Figures 5(d) and 5(e)). It is reported that *α*-SMA-labeled myofibroblasts contribute substantially to fibrosis progression [24]. Fibrosis and *α*-SMA immunostaining cells colocalized around bile ducts in the current study (Figures 5(a) and 5(b)). The percentages of *α*-SMA-stained area in the BDI group and PPVA + BDI groups were significantly increased (Figure 5(f)). However, *α*-SMA immunoreactivity was significantly reduced by PPVA in the PPVA + BDI group in comparison with the BDI group.

It is reported that PVA with unrestricted inflow induces hepatic fibrosis [25]. To investigate whether the fibrosis and ductular reaction found in the PPVA + BDI group were caused by PV hypertension or anastomosis occlusion, the patency of the anastomosis, blood inflow, and PV pressure were examined. Ultrasonography confirmed the anastomosis patency in the PPVA and PPVA + BDI groups (data not shown). However, PV hypertension induced by PPVA was found on day 1; the PV pressure had decreased to near normal levels on day 14 (Figure 5(g)).

### 3.6. PPVA Improved BDI-Induced Hypoxia and Hypoxia-Associated Gene Changes

**Figure 6:**
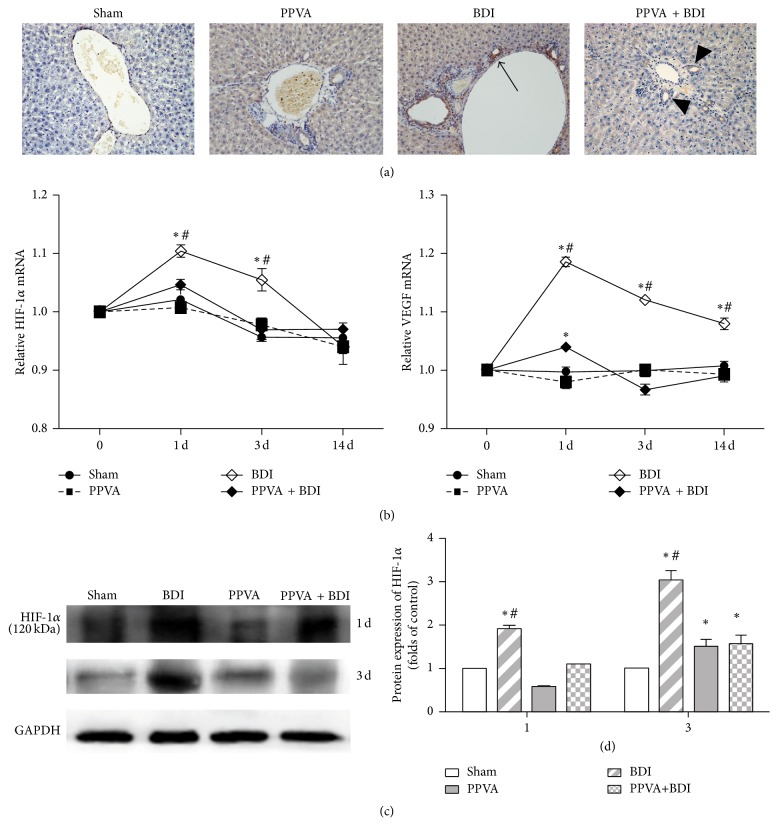
PPVA improved bile duct hypoxia after hepatic arterial deprivation. (a) Representative HIF-1*α* immunostaining images are shown. Karyotypic HIF-1*α* expression (arrow) in the BDI group and cytoplasmic HIF-1*α* expression (arrowheads) in the PPVA + BDI group were detected in the bile duct cells 1 day after the surgery (magnification, 200x). (b) Hepatic HIF-1*α* and VEGF mRNA expression was analyzed using qRT-PCR. (c) Total protein extracts were subjected to western blotting. Representative gels showed that BDI increased HIF-1*α* protein expression, which was reduced by PPVA. PCR results are expressed as the mean ± SEM of at least triplicate measurements. Western blotting data are the mean ± SEM of three independent experiments and are normalized to GAPDH. ^*∗*^ *P* < 0.01 versus the Sham group; ^#^ *P* < 0.01 versus the PPVA + BDI group. Differences were assessed by two-way ANOVA and comparisons between groups were performed by Bonferroni posttests at each time point.

On immunostaining, karyotypic HIF-1*α* expression in the BDI group and cytoplasmic HIF-1*α* expression in the PPVA + BDI group were detected in the bile duct cells 1 day after surgery (Figure 6(a)). This finding indicated the presence of bile duct hypoxia and indicated that the hypoxia was more severe in the BDI group [26]. Low HIF-1*α* expression in the perivenular hepatocytes was occasionally detected in all groups, except for the Sham group, on day 1. HIF-1*α* and VEGF mRNA expression were significantly higher in the BDI group than other groups. These expressions showed a tendency to decrease by day 14 (Figure 6(b)). Consistent with the qRT-PCR results, western blot analysis showed that BDI increased HIF-1*α* protein expression, which was attenuated by PPVA (Figure 6(c)).

### 3.7. PPVA Improved BDI-Induced Cholestasis and Inflammation

**Figure 7:**
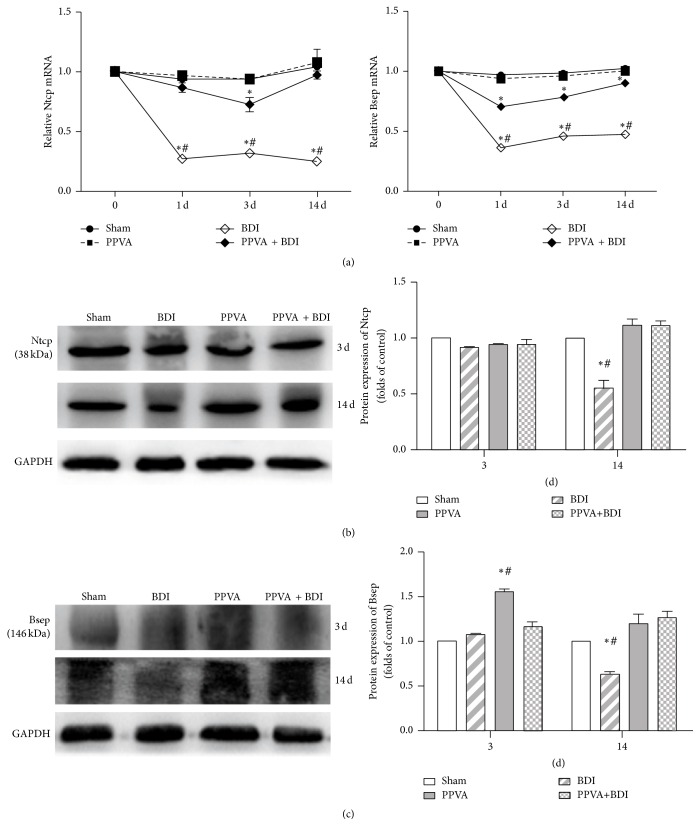
PPVA improved BDI-induced cholestasis. (a) Ntcp and Bsep mRNA expression was analyzed using qRT-PCR. Ntcp protein expression (b) and Bsep protein expression (c) were measured by western blotting in all groups. PCR results are expressed as the mean ± SEM of at least triplicate measurements. Western blotting data represent the mean ± SEM of three independent experiments and are normalized to GAPDH. ^*∗*^ *P* < 0.01 versus the Sham group; ^#^ *P* < 0.01 versus the PPVA + BDI group. Differences were assessed by two-way ANOVA and comparisons between groups were performed by Bonferroni posttests at each time point.

Bile salt uptake from the portal blood into the hepatocytes by Ntcp largely depends on the energy from the sodium gradient maintained by the ATP-dependent sodium pump (Na+/K+-ATPase) [27]. Bile salts are exported into the canaliculus by ATP-dependent Bsep, which is the rate-limiting step of hepatocellular bile salt transport [27]. Therefore, the ongoing energy supply of ATP is critical for transcellular bile salt transportation. Consistent with the abovementioned ATP changes, Ntcp and Bsep gene expression were significantly decreased in the BDI group. Similar changes, but to a lower extent, were found in the PPVA + BDI group (Figure 7(a)). Protein expression of Ntcp and Bsep was also significantly reduced 14 days after surgery in the BDI group. However, changes of protein expression of Ntcp and Bsep had no statistical differences in the BDI group on postoperative day 3 in comparison with the Sham group and the PPVA + BDI group (Figures 7(b) and 7(c)), which were not consistent with the changes of the mRNA levels of Ntcp and Bsep. There were no statistical differences in both gene and protein expression levels of Ntcp and Bsep in the PPVA + BDI group compared with the Sham group at day 3 and day 14.

**Figure 8:**
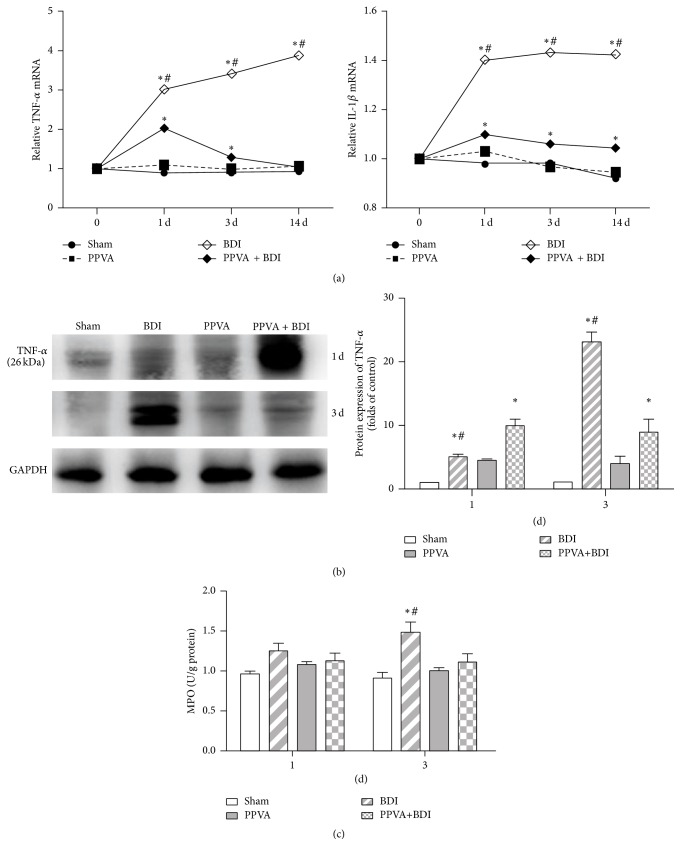
PPVA improved BDI-induced inflammation. (a) TNF-*α* and IL-1*β* mRNA expression was analyzed using qRT-PCR. (b) TNF-*α* protein expression, which was evaluated by western blotting, was consistent with the qRT-PCR results. (c) Changes in myeloperoxidase (MPO) activity, a marker for neutrophil recruitment, were observed in all groups. PCR results are expressed as the mean ± SEM of at least triplicate measurements. Western blotting data represent the mean ± SEM of three independent experiments and are normalized to GAPDH. ^*∗*^ *P* < 0.01 versus the Sham group; ^#^ *P* < 0.01 versus the PPVA + BDI group. Differences were assessed by two-way ANOVA and comparisons between groups were performed by Bonferroni posttests at each time point.

TNF and IL-1*β* gene expression were significantly elevated in the BDI and PPVA + BDI groups. However, both were lower in the PPVA + BDI group than in the BDI group (Figure 8(a)). TNF-*α* protein expression was consistent with the qRT-PCR results (Figure 8(b)). However, surprisingly this expression was significantly raised in the PPVA + BDI group on day 1, possibly due to the PPVA procedure itself. Likewise, MPO activity, a neutrophil marker, was higher in the BDI group on day 3 and was significantly reduced by PPVA (Figure 8(c)).

## 4. Discussion

The principal finding from this study is that complete dearterialization of the rat liver led to severe bile duct injury, including bile leakage, bile infarcts, and biloma formation. PPVA attenuated the ischemic damage to cholangiocytes by improving the energy state, attenuating cholestasis and reducing proinflammatory cytokine production, ductular reaction, and fibrosis.

It has been demonstrated that HA ligation alone does not induce ischemic liver injury and bilomas in rodents [4, 5]. The mechanisms of the hepatobiliary damage induced by complete hepatic deprivation in the present study are complex and multifactorial. First, cholangiocytes were the primary target of the initial insult, hepatic arterial ischemia [2]. Evidence of BDI and injury has been presented in this current study and a previous study [13]. Similarly, hepatocytes were also damaged by hypoxia-ischemia due to their high metabolic rate and abundant mitochondria. These changes were predominantly attributable to ischemia-hypoxia. The cholestatic changes detected here were also believed to result from ischemia-hypoxia [5], because the Ntcp and Bsep gene expression were decreased as early as on postoperative day 1 prior to the biochemical changes, in accordance with the ATP changes. In the absence of bile duct obstruction, these changes in bile transporters were not adaptive responses to biliary obstruction but consequences of ischemia-hypoxia [5, 19].

Second, hypoxia-induced cholestasis further sensitizes the liver to ischemic injury [28–30] and contributes to hepatobiliary injury through special mechanisms. Retention of bile acids and bilirubin in hepatocytes is toxic to the liver, inhibits the respiratory chain, and decreases ATP synthesis [31]. More importantly, bile acid-mediated toxicity alters mitochondrial membrane permeability, leading to mitochondrial dysfunction [31, 32]. In addition to bile retention, bile extravasation was detected after BDI in the present study. Bile effusion leads to portal inflammation by stimulating cytokine and chemokine release from injured bile ducts, macrophages, and lymphocytes [33]. Absorption of the bile constituents into the blood leads to bile infarcts in the absence of biliary obstruction [34]. Along with these deleterious events, bile constituents infiltrate the liver parenchyma, leading to liver necrosis and finally bilomas, which may evolve into liver abscesses after infection. Our results showed that there were poor correlations between the level of mRNA and the level of protein expression of Ntcp and Bsep on postoperative day 3. This discrepancy can be explained that bile transporter proteins may differ largely in their in vivo half-lives and some complicated posttranscriptional mechanisms are yet to be fully understood [35]. However, our results demonstrated that PPVA attenuated these deleterious effects by improving cholestasis 14 days after surgery.

It is worth noting that theoretically the bile duct cannulation used here might result in biliary obstruction, which contributes to liver damage. Interestingly, previous studies demonstrated that bile duct cannulation in this BDI model does not result in obstructive changes. Thus biliary obstruction is not one of the mechanisms for liver injury observed in the current study. However, transient partial biliary obstruction in some rats induced by edema and inflammation of the CBD cannot be completely excluded due to technique limitations.

Generally, bile leaks from the ruptured biliary system; however, we failed to locate the exact rupture site in the bile ducts, as sampling the entire biliary system is a formidable task. The tight junctions of hepatocytes are less tight than those of cholangiocytes [36]. Hence, bile may have extravasated from necrotic bile canaliculi or ductules or from tight junctions between hepatocytes due to increased permeability [36]. This possibility was supported by our findings and the fact that the PBP is absent in the areas of terminal cholangioles and the canals of Hering, which are more susceptible to ischemia-hypoxia than larger bile ducts [37].

Third, cross-talk interactions of ischemia-hypoxia and cholestasis lead to persistent inflammation, a significant contributory factor to ischemic cholangiopathy. For example, TNF-*α* and IL-1*β* play important roles in cholestasis-induced cell death and fibrosis [32, 33]. Inflammation further aggravates cholestasis. Moreover, ischemia-hypoxia can directly induce inflammation [38] through HIF-1*α* secretion by activated hepatic stellate cells or Kupffer cells [39]. Inflammation enhances the effects of HIF-1*α*, which plays a critical role in inflammation, angiogenesis, and fibrosis [39, 40]. Taken together, ischemia-hypoxia, cholestasis, and inflammation form a vicious cycle, act concomitantly to perpetuate the initial insult of hepatic arterial ischemia, and, consequently, lead to severe liver damage. These mechanisms of pure ischemia in the current study are different from those of ischemia-reperfusion injury in liver transplantation [30, 32], in which the liver also undergoes complete dearterialization.

Finally, ductular reaction and periductal fibrosis are possible mechanisms underlying bile duct injury. The increased oxygen and nutritional demands of proliferated bile ducts may further aggravate ischemia-hypoxia [41]. Moreover, collagen deposition around proliferated bile ducts, found in this study, could separate the PBP from the bile ducts [20, 33], further impeding nutrition and oxygen exchange.

PPVA attenuated acute ischemic injury to cholangiocytes by improving hypoxia, cholestasis, and inflammation and consequently disrupting the vicious cycle to some extent. However, unlike its satisfactory effects on the liver parenchyma, PPVA alleviated ischemic bile duct injury and yet did not fully compensate for the loss of the hepatic arterial blood supply. This may be due to the back-diffusion of blood from arterioportal shunts, which have an abnormal oxygen gradient; thus, this blood is inadequate to satisfy the oxygen needs of cholangiocytes [37]. In particular, unlike hepatocytes, which receive dual blood supply, cholangiocytes entirely depend on the hepatic arterial blood supply for oxygenation.

Our data partly account for the development of hepatic infarcts or liver abscesses in the native liver after hepatic arterial disruption in clinical practice [8]. Understanding the mechanisms responsible would be useful for designing optimal therapy. PPVA is beneficial for hypoxic and cholestatic liver injury when hepatic arterial reconstruction is technically arduous or unfeasible after the HA has been incidentally ligated or intentionally resected during hepatobiliary surgery.

PPVA has its limitations. Our results indicated that PPVA aggravated hepatocyte injury on postoperative day 1, due to the longer operating time and temporary PV occlusion during anastomosis, which transiently elevated the PV pressure. In the clinical setting, PV flow can be controlled without total occlusion. The risk of portal hypertension can be reduced by anastomosing the PV with a small-caliber artery, such as the inferior mesenteric artery, ileal branch of the superior mesenteric artery, and the right gastroepiploic artery [16]. Finally, only the short-term effects of PPVA were examined; its long-term effects require further investigation.

In conclusion, complete hepatic deprivation in rats results in severe ischemic damage, including bile infarcts and bilomas, which may evolve into liver abscesses after infection. The current findings suggest that PPVA has a beneficial effect on ischemia-hypoxia-induced bile duct injury.

## Figures and Tables

**Table 1:** Primers used in this study (*Rattus norvegicus*).

Gene	Forward 5′-3′	Reverse 5′-3′
*HIF-1α*	TCAGTTGTCACCATTAGAGAGCAAT	GGGTCTGCTGGAATCCTGTAAC
*VEGF*	GGGCCTCTGAAACCATGAACT	TGGTGGAGGTACAGCAGTAAAGC
*Bsep*	TGCCAAGGATGCTAATGCATAC	TCATCTGGCCTCCTCCTTCTC
*Ntcp*	TCTGCTCTCTTCCAACTCAATCC	GAGTTGAATGTTTTGGAATCCTGTT
*TNF-α*	ACAAGGCTGCCCCGACTAC	CTCCTGGTATGAAATGGCAAATC
*Il-1β*	AGGAGTGCCGGCGTTTC	CTGGAGCCATCTCGGTTCA
*Procollagen 1*	GAGAGTACTGGATCGACCCTAACC	CTGACCTGTCTCCATGTTGCA
*Actin*	TCCTGACCCTGAAGTATCCGATA	GGTGCCAGATCTTTTCCATGTC
